# Supplementary material for: Optimal input DNA thresholds for genome skimming in marine crustacean zooplankton
Source: PeerJ. 2025 Feb 26;13:e19054. doi: 10.7717/peerj.19054 (PMC11871894; doi:10.7717/peerj.19054)
Supplement: Supplemental Information 1 — *sample without FX enhancer for fragmentation. [file peerj-13-19054-s001.docx]

|  | Fragmentation | Adaptor | Amplification |
| --- | --- | --- | --- |
|  | time (min) | dilution | PCR cycles |
| 10 ng | 24* | 1:10 | 10 |
| 1 ng | 14 | 1:10 | 12 |
| 100 pg | 20 | 1:100 | 17 |
| 10 pg | 25 | 1:1000 | 20 |
| 1 pg | 30 | 1:1000 | 23 |
