# Supplementary material for: Optimal input DNA thresholds for genome skimming in marine crustacean zooplankton
Source: PeerJ. 2025 Feb 26;13:e19054. doi: 10.7717/peerj.19054 (PMC11871894; doi:10.7717/peerj.19054)
Supplement: Supplemental Information 2 — HTS, high-throughput sequencing. [file peerj-13-19054-s002.docx]

| Species | HTS | Input DNA | Raw reads | Filtered reads |
| --- | --- | --- | --- | --- |
| *Euphausia pacifica* | HiSeq | 10 ng | 2,765,331 | 2,026,924 |
|  |  | 1 ng | 4,858,336 | 3,714,518 |
|  |  | 100 pg | 7,178,356 | 5,567,958 |
|  |  | 10 pg | 6,211,455 | 4,421,275 |
|  |  | 1 pg | 9,975,882 | 6,026,651 |
| *Calanus glacialis* | NovaSeq | 10 ng | 8,213,924 | 6,436,276 |
|  |  | 1 ng | 6,963,873 | 5,564,122 |
|  |  | 100 pg | 8,586,159 | 6,380,746 |
|  |  | 10 pg | 1,580,174 | 1,103,249 |
|  |  | 1 pg | 419 | 106 |
