# Supplementary figures and images for: Optimal input DNA thresholds for genome skimming in marine crustacean zooplankton
Source: PeerJ. 2025 Feb 26;13:e19054. doi: 10.7717/peerj.19054 (PMC11871894; doi:10.7717/peerj.19054)

A

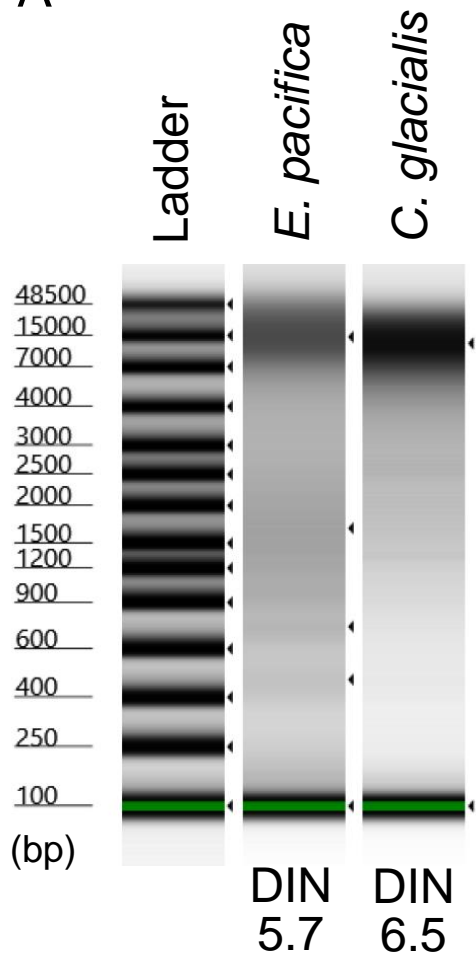

B

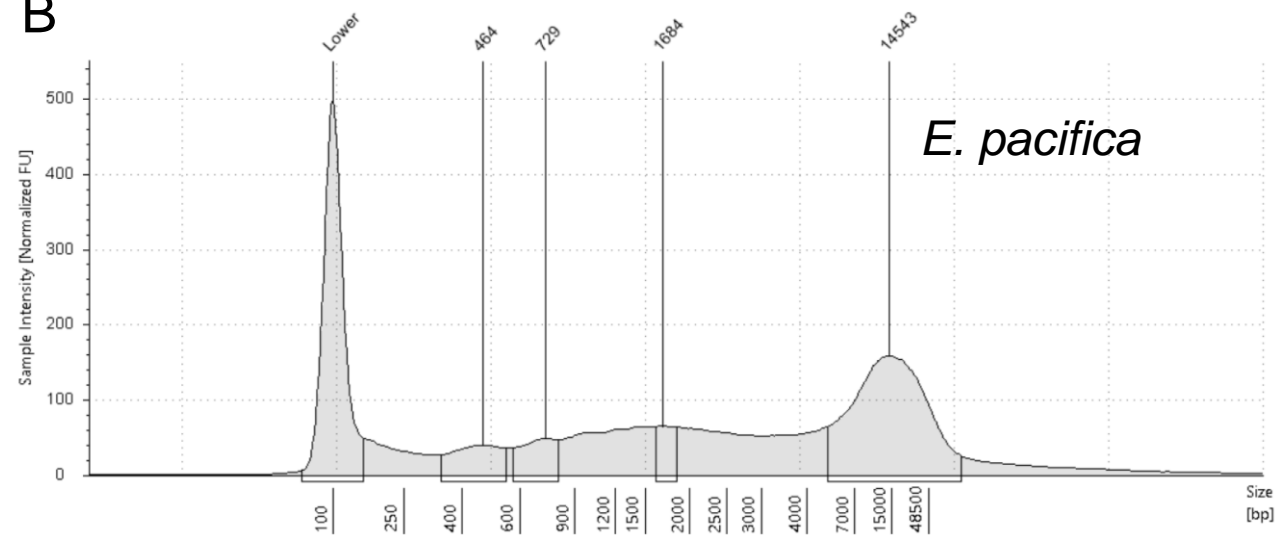

C

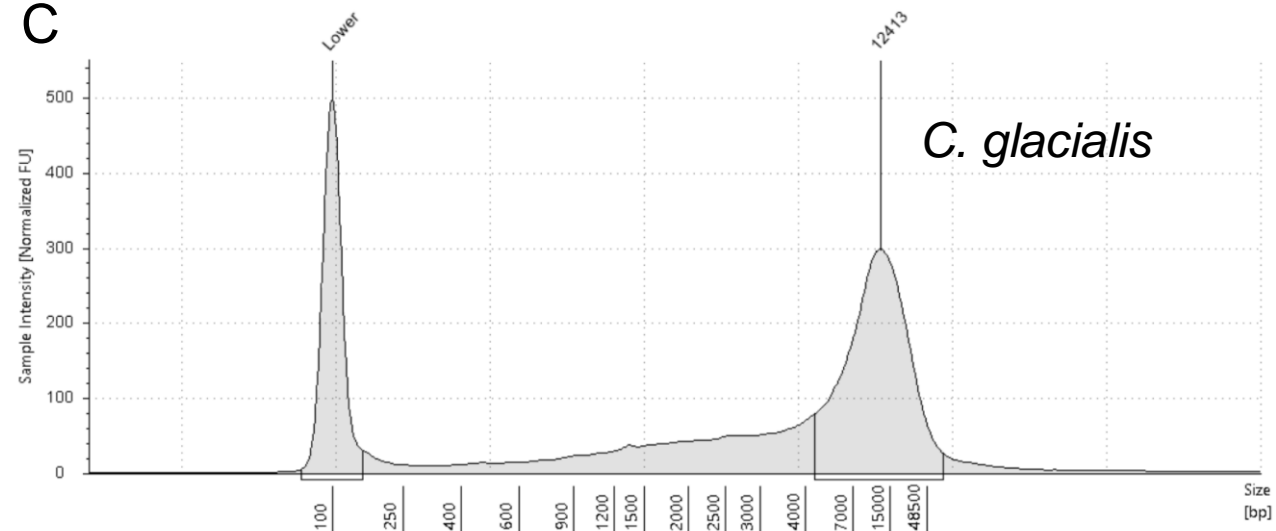

Supplement: Supplemental Information 3 — (A) Gel electrophoresis image and DIN for DNA from Euphausia pacifica and Calanus glacialis. (B) Electropherogram for DNA size (bp) in E. pacifica. (C) Electropherogram for DNA size (bp) in C. glacialis. [file peerj-13-19054-s003.pdf]
